# Supplementary material for: Saccharomyces boulardii (CNCM I-745) improves intestinal damage in sepsis by remodeling intestinal flora structure
Source: Front Cell Infect Microbiol. 2025 Jul 31;15:1602792. doi: 10.3389/fcimb.2025.1602792 (PMC12350329; doi:10.3389/fcimb.2025.1602792)
Supplement: Supplementary file 1 [file DataSheet1.zip › summary/summary/8_Advanced_analyse/2_PICRUSt2_result/KEGG/Group.BW4_vs_BW0/PICRUSt2_KEGG_level3_Group.BW4_vs_BW0_diff.pdf]

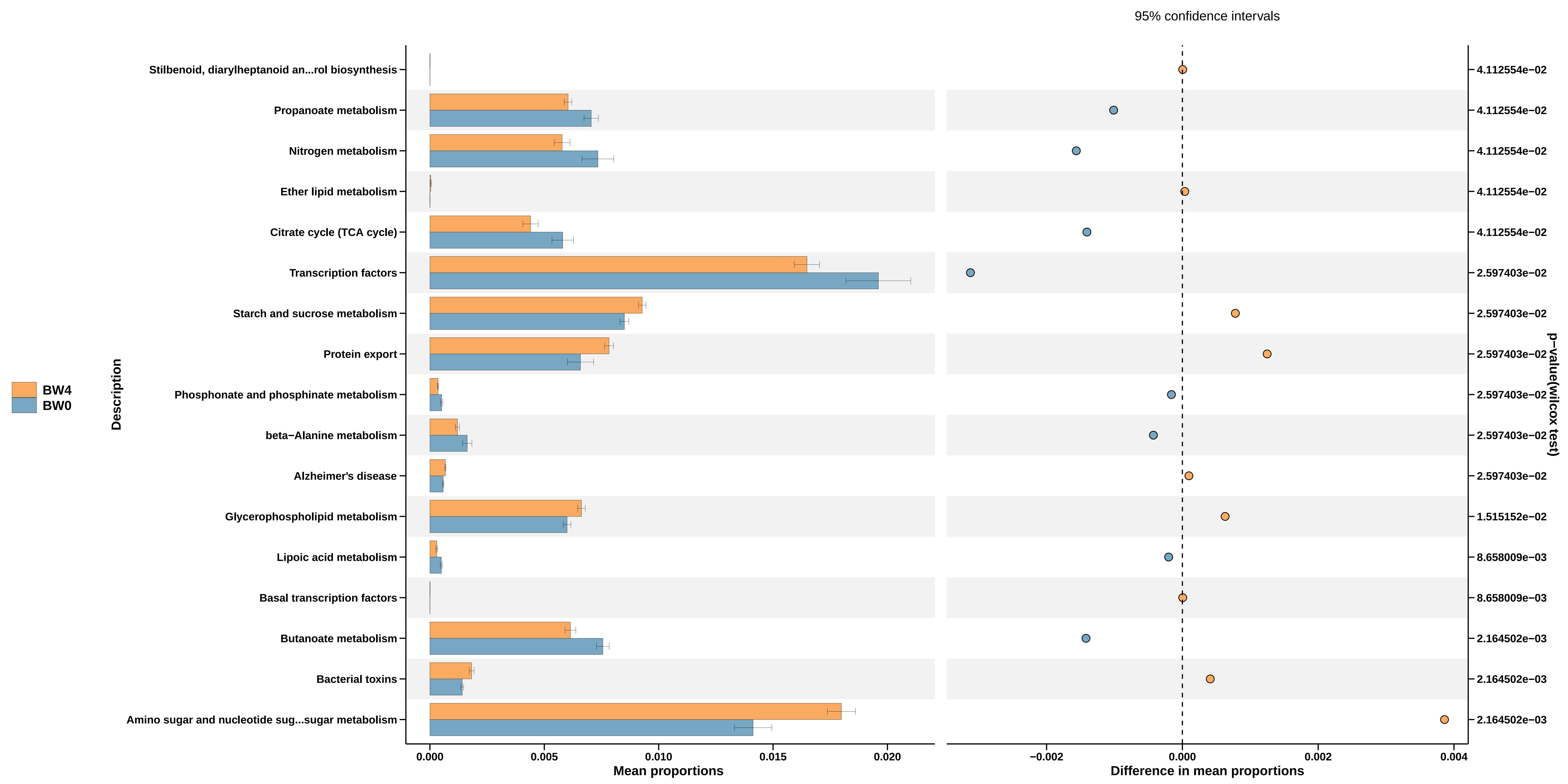

95% confidence intervals
Stilbenoid, diarylheptanoid an...rol biosynthesis
Propanoate metabolism
Nitrogen metabolism
Ether lipid metabolism
Citrate cycle (TCA cycle)
Transcription factors
Starch and sucrose metabolism
Protein export
Phosphonate and phosphinate metabolism
beta−Alanine metabolism
Alzheimer's disease
Glycerophospholipid metabolism
Lipoic acid metabolism
Basal transcription factors
Butanoate metabolism
Bacterial toxins
Amino sugar and nucleotide sug...sugar metabolism
4.112554e−02
4.112554e−02
4.112554e−02
4.112554e−02
4.112554e−02
2.597403e−02
2.597403e−02
2.597403e−02
2.597403e−02
2.597403e−02
2.597403e−02
1.515152e−02
8.658009e−03
8.658009e−03
2.164502e−03
2.164502e−03
2.164502e−03
BW4
BW0
Description
0.000
0.005
0.010
0.015
0.020
Mean proportions
−0.002
0.000
0.002
0.004
Difference in mean proportions
p−value(wilcox test)
